# Contribution of cuproptosis and Cu metabolism‐associated genes to chronic obstructive pulmonary disease

**DOI:** 10.1111/jcmm.17985

**Published:** 2023-10-06

**Authors:** Wenchuan Qi, Lu Liu, Qian Zeng, Ziyang Zhou, Daohong Chen, Bin He, Siyao Gong, Lei Gao, Xiao Wang, Jian Xiong, Dingjun Cai, Shuguang Yu, Ling Zhao

**Affiliations:** ^1^ Acupuncture and Tuina School Chengdu University of Traditional Chinese Medicine Chengdu Sichuan China; ^2^ Acupuncture & Chronobiology Key Laboratory of Sichuan Province Chengdu Sichuan China

**Keywords:** bioinformatics, chronic obstructive pulmonary disease, copper metabolism‐related genes, cuproptosis

## Abstract

Airway epithelial cell injury plays a crucial role in the pathogenesis of chronic obstructive pulmonary disease (COPD). However, a novel form of Cu‐induced programmed cell death known as cuproptosis has not yet been thoroughly investigated in the context of COPD. Clinical reports have suggested that high copper exposure may increase the risk of COPD. In this study, we aimed to determine the expression and potential functions of cuproptosis‐related genes and genes associated with copper metabolism in COPD. We initially identified 52 copper metabolism‐related genes based on a review of the literature. Subsequently, we calculated the expression levels of these genes using data from four GEO datasets. To gain insights into the activated signalling pathways and underlying mechanisms in COPD patients, we conducted Gene Ontology (GO) and KEGG pathway analyses, examined protein–protein interactions, and performed weighted correlation network analysis. Our findings revealed that 18 key copper metabolism‐related genes, including 5 cuproptosis‐related genes, were significantly enriched in signalling pathways and biological processes associated with the development of COPD. Further analysis of clinical data and animal experiments confirmed the high expression of certain cuproptosis key regulators, such as DLD and CDKN2A, in both healthy smokers and COPD smokers. Additionally, these regulators exhibited abnormal expression in a COPD rat model. Notably, copper content was found to be elevated in the lung tissues of COPD rats, suggesting its potential involvement in cuproptosis. These findings provide an experimental foundation for further research into the role of cuproptosis in COPD. Targeting copper metabolism‐related genes may represent an effective approach for the treatment of COPD.

## 1 INTRODUCTION

In chronic obstructive pulmonary disease (COPD), persistent airflow obstruction is caused by high exposure to toxic gases and particles, such as cigarette smoke.^1^, ^2^ COPD is a progressive condition characterized by emphysema, chronic bronchitis and small airway obstruction, and is evidenced by dyspnoea, cough and sputum production. COPD has become a leading cause of disability and is the third most common cause of death worldwide,^3^, ^4^ accounting for 3.23 million deaths in 2019. Major risk factors for COPD are outdoor and indoor air pollution (e.g. biofuels utilized for heating and cooking), occupational dust, chemicals and especially, smoking.^5^, ^6^, ^7^ Although COPD pathogenesis is unclear, its hallmarks are oxidative stress,^8^ immunity and inflammation,^9^ apoptosis,^10^ metaplastic epithelial damage,^11^ mucus hypersecretion^12^ and fibrotic airways.^13^

In COPD patients, diminished lung function has been correlated with high levels of heavy metals.^14^ Long‐term exposure to copper (Cu) and iron (Fe) in PM2.5 was linked to increased lung reactive oxygen species (ROS) concentration and heightened incidence of respiratory disease, indicating the adverse effect of non‐tailpipe emission on the respiratory system.^15^ Also, the severity of COPD exacerbation was associated with elevated levels of zinc (Zn), Cu and lipid peroxidation (malondialdehyde, MDA); in COPD patients, serum Cu and MDA concentration were higher than in the controls,^16^ whereas serum paraoxonase (PON1) activity was lower. Comparing COPD smokers and non‐smokers, Zn and Cu/Zn ratios were not statistically different, but differences were observed in Cu, MDA and serum PON1 activity.^17^

Heavy metals can induce regulated cell death through various mechanisms. For example, intracellular Cu accumulation can induce cuproptosis, a unique type of programmed cell death, by triggering mitochondrial fatty‐acylated protein aggregation and S‐Fe cluster protein destabilisation.^18^ In that work, it was shown that Cu toxicity is highly correlated with mitochondrial activity. Mitochondrial dysfunction is a critical event in the pathogenesis of COPD.^19^ Stress factors including cigarette smoke and other environmental pollutants induce the formation of free radicals, leading to oxidative stress, in which mitochondria plays a critical role as it regulates the production of superoxide radicals.^8^ Cigarette smoke exposure causes mitochondrial dysfunction/mitochondrial autophagy, resulting in perinuclear mitochondria accumulation in fibroblasts and human lung epithelial cells.^20^ It has been reported that cigarette smoke leads to mitochondrial dysfunction, and reversing mitochondrial functions is effective in different COPD models.^21^ As a result, mitochondrial processes and pathways are gaining increasing importance as potential drug targets for COPD. Currently, the lack of understanding of cuproptosis and copper metabolism in the pathogenesis of COPD may be limiting the treatment options for this disease. Furthermore, the underlying mechanisms of cuproptosis and the changes in copper metabolism‐associated genes during disease progression remain elusive. In this study, through the use of bioinformatics analysis of clinical data and animal experiments, we have uncovered insights into cuproptosis and identified copper metabolism‐associated genes that may contribute to the development of COPD.

## 2 MATERIALS AND METHODS

### 2.1 Data acquisition and preprocessing

We obtained gene expression data from four gene array expression Series Matrix Files of small airway epithelium specimens available in the GEO datasets (https://www.ncbi.nlm.nih.gov/geo/). These datasets, namely GSE5058, GSE8545, GSE11906 and GSE20257, comprised 15, 18, 33 and 23 COPD samples, respectively, as well as 24, 36, 98 and 112 healthy control samples, respectively. The experiments were conducted using the Affymetrix Human Genome U133 Plus 2.0 Array (GPL570 platform). To process the data, we obtained the gene symbols of the datasets with annotation packages and utilized R software. We standardized the mRNA expression data using the limma package version 3.40.6 and R version 4.13. Given the small sample size of small airway epithelium specimens in COPD, we merged and batch‐normalized GSE8545, GSE11906, GSE5058 and GSE20257 into a single group.

### 2.2 Selection of Cu metabolism‐associated genes between COPD and healthy control groups

A total of 52 widely recognized Cu metabolism‐associated genes were collated from the published list shown in Table S1. The mRNA expression data was obtained from the merged and batch‐normalized group. Differential expression analysis for these genes was conducted with wilcox_test. The correlation among cuproptosis‐associated genes was identified with the corrplot R package. *p* < 0.05 was deemed statistically significant.

### 2.3 Weighted gene co‐expression network analysis (WGCNA) analysis

We performed WGCNA to identify co‐expressed gene modules and investigate the relationship between gene network and phenotype. The network type was set to ‘unsigned’. To establish the co‐expression network, we utilized the topological overlap matrix with an appropriate soft thresholding parameter which helps determine connectivity and dissimilarity among genes. We employed the dynamic hybrid cut method, a bottom‐up algorithm, to identify co‐expressed gene modules. This method involved constructing a hierarchical clustering tree in which each leaf represented a single gene. Genes with similar expression data or functions were clustered closely, forming branches in the tree that represented gene modules. To assess the relationship between these modules and the phenotype, we used Pearson's test to calculate the correlation between module eigengenes (MEs) and the phenotype. A module was considered significantly related to the phenotype when the *p*‐value was less than 0.05.

### 2.4 Gene enrichment analysis

Gene ontology (GO) and Kyoto Encyclopedia of Genes and Genomes (KEGG) were used to assess the biological roles of the prognostic candidates by the ‘clusterProfiler’ R package. The Benjamini−Hochberg method for the multiple correction, and a false discovery rate (FDR) < 0.05 was considered to be of significance.

### 2.5 PPI (Protein–Protein Interaction) networks of Cu metabolism‐associated genes with key COPD genes

The interactions among 18 Cu metabolism‐associated genes and Cu metabolism‐associated genes with key COPD genes (Table S2) obtained from literature were analysed by PPI network, and the results revealed that DLD and CDKN2A were identified as one of core genes in the Cu metabolism process of COPD. *p* < 0.05 was deemed statistically significant.

### 2.6 Rat models and cigarette smoke exposure protocol

Sprague–Dawley rats (male, SCXK [Chuan] 2015‐030; Dashuo) were purchased and maintained in the animal facility at the Chengdu University of TCM, with 7‐day acclimatisation period, following a 12‐h dark/light cycle. All animal protocols were approved by Chengdu University of TCM of Animal Care Committee. COPD was induced by cigarette smoke (CS) inhalation (Jiaozi) and LPS injection. A whole‐body exposure system was used (CSM‐100C, Tow‐Int Tech) within a barrier facility. Rats (*n* = 10 per group) were exposed to CS from 15 cigarettes of Jiaozi (11 mg tar and 1.1 mg nicotine; Sichuan Tobacco Industry) for 60 min, twice a day (30 cigarettes daily) for 12 weeks. LPS (L2880, Sigma) intervention (1 mg/mL, 0.2 mL) was implemented every 30 days (three times for 12 weeks).^22^ Pulmonary function was assessed with a rat pulmonary function measurement system (AniRes2005, Beilanbo) to confirm COPD.

### 2.7 Lung function examination and content copper in lung tissue

Lung function in rats was assessed using the AniRes2005 lung function system (Bestlab Company), as directed by the manufacturer. The rats were anesthetized with 3% pentobarbital sodium (40 mg/kg) (Sigma), after exposing the trachea and intubation. They were placed in a volume tracing box with a signal conditioner, a ventilator and entrotracheal intubation, which sent data to the computer, including airflow and changes in lung volume. The breathing ratio was set to 20:10 and the respiratory rate of each rat was 70 times/min. The following evaluation indicators were used: forced expiratory volume in 0.1 s (FEV 0.1) and 0.3 s (FEV 0.3), and the ratio of FEV 0.1 or FEV 0.3 to forced vital capacity (FEV 0.1/FVC or FEV 0.3/FVC). The assays were conducted using the Copper Assay Kit (E010‐1‐1; Nanjing Jiancheng Institute of Bioengineering) according to the kit's protocols.

### 2.8 Lung tissue histopathology

After the rats were sacrificed, lung tissue specimens were fixed with formalin neutral buffer solution (10%), embedded in paraffin, sectioned at 4‐mm thickness and used for haematoxylin–eosin staining. The degree of damage, for example, alveolar congestion, inflammatory cell infiltration and widening of alveolar septum, was evaluated with reference to the scoring standard of pathological damage of lung tissue from literature,^23^ and scored according to severe injury (3 points), moderate injury (2 points), mild injury (1 point) and no injury (0 points). Each group selected six rats for scoring, taking the average value as the final score. Detection of goblet cells in bronchi of COPD rats by PAS staining,^24^ and average optical (AO) value was used to judge the expression level of goblet cells (*n* = 6 in each group).

### 2.9 Real‐time quantitative PCR (RT‐qPCR)

The lung tissue of each rat was removed and quickly frozen. Total RNA extraction was performed by RNAprep Pure Tissue Kit (TIANGEN). Purity and concentration of RNA in the extract were assessed by gel electrophoresis and spectrophotometry. RNA (1 μg) was reverse‐transcribed into cDNA (TIANGEN). The PCR reaction mixture contained 10 μL cDNA, 10 μL SyberGreen Mix (TIANGEN) and 0.5 μL forward/reverse primers each, yielding a total volume of 20 μL. RT‐qPCR was conducted on a BIOER Detection System (Bioer Technology, Hangzhou, China) as follows: predegeneration for 3 min at 95°C, degeneration for 30 s at 95°C, reannealing for 30 s at 55°C and 40 extension cycles for 30 s at 72°C. After PCR, the products were subjected to relative quantitative evaluation using the 2^−△△Ct^ method. GAPDH was used as an intern control. The RT‐qPCR primer pair was designed by Tsingke, Chengdu and the primer sequences are as follows:

DLD F1 AGAGTCTGCCATGCACATCC

DLD R1 ATGTCGAATCTCGTAGACCGT

CDKN2A F1 GCGTTGCCAGAAGTGAAGCCA

CDKN2A R1 CGTCGTGCGGTATTTGCGGTAT

GAPDH F1 GATGCTGGTGCTGAGTATGRCG

GAPDH R1 GTGGTGCAGGATGCATTGCTCTGA

### 2.10 Western blotting

Lung tissues were lysed in RIPA buffer (Servicebio, G2002) consisting of a protease inhibitor cocktail (Solarbio, P6730), or lysed with Laemmli sample buffer. Mitochondrial protein separation was performed using a mitochondrial protein isolation kit (Beijing Solarbio Science & Technology). For each experiment, proteins were separated through SDS‐PAGE. After SDS‐PAGE, the separated proteins were transferred onto PVDF membranes and subsequently exposed to primary antibodies overnight at 4°C. The primary antibodies used were as follows: β‐actin (ZEN BIO, 200068‐8F10) at a dilution of 1:5000, DLD (Fntest, FNab02406) at a dilution of 1:1000 and CDKN2A (Immunoway, YM0494) at a dilution of 1:1000. After washing several times with PBST (phosphate‐buffered saline with Tween 20), membranes were exposed to secondary antibody for 60 min. Detection of proteins using chemiluminescence. Results were analysed with Image J version V1.8.0.112 (NIH).

## 3 RESULTS

### 3.1 Expression of cuproptosis and copper metabolism‐associated genes in COPD

**FIGURE 1:**
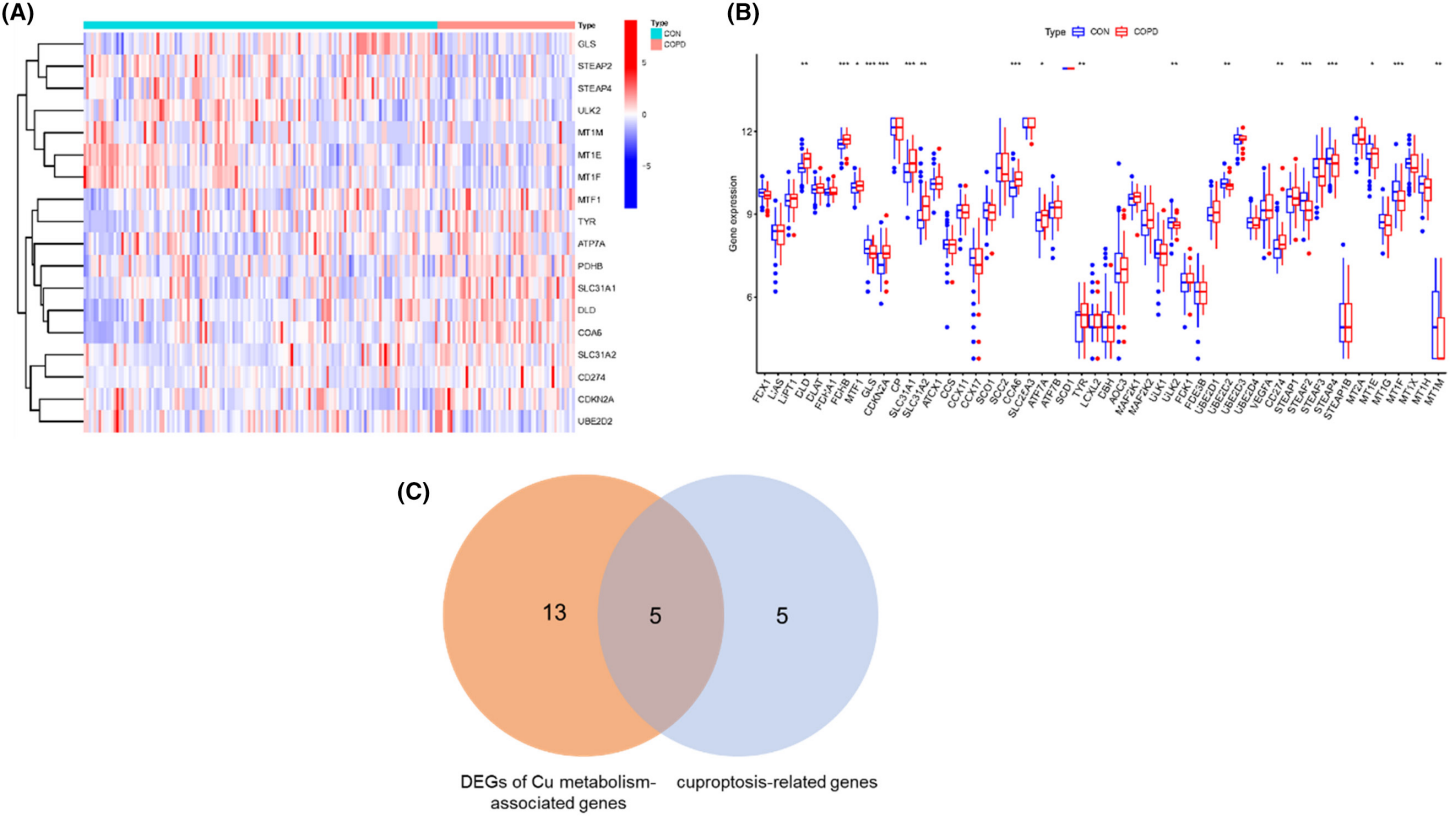
Expression of Cu metabolism‐associated genes in COPD patients and control subjects. (A, B) Heatmap (A) and histogram (B) of 52 copper metabolism‐related genes (COPD vs control); (C) 5 copper metabolism‐related genes and cuproptosis‐related genes were shared.

The analysis initially focused on assessing the differential expression of 52 cuproptosis and Cu metabolism‐associated genes in both COPD and control samples. Within the COPD group, a total of 18 differentially expressed genes (DEGs) were identified (see Figure 1A,B). Notably, among these DEGs, five genes have been previously reported to be associated with cuproptosis^18^ (see Figure 1C). Overall, these findings demonstrate that cuproptosis and copper metabolism‐related genes are responsible for COPD disease.

### 3.2 PPI network analysis of copper metabolism‐associated genes

**FIGURE 2:**
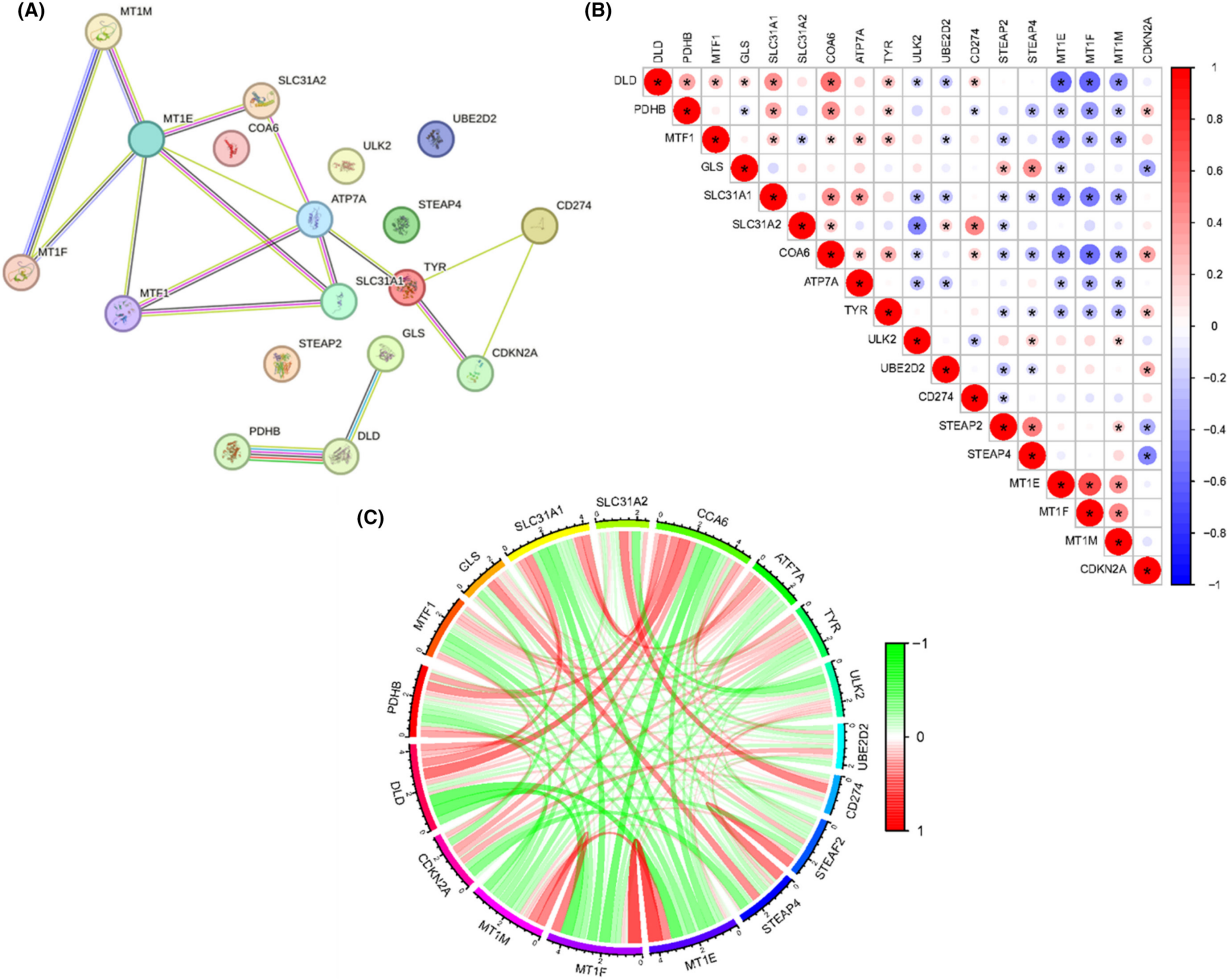
Correlation between the expression of copper metabolism‐associated genes. (A) PPI analysis among 18 copper metabolism‐related genes; (B) correlation among the expression of 18 Cu metabolism‐associated genes; (C) Circos plot displaying the interconnectivity among genes related to 18 copper metabolism‐related genes. Thickness and colour of the ribbons correspond to the correlation of genes expression.

To determine the interactions between the 18 copper metabolism‐related genes in COPD disease, we performed a PPI network analysis. These genes frequently interacted (Figure 2A), suggesting they cooperate. Among them, MTE1 interacts more closely with other proteins. Thus, we further investigated the expression correlation of these genes. Highly consistent correlations were observed between regulators of cuproptosis (Figure 2B,C); for example, DLD, PDHB, MTF1, GLS or CDKN2A were significantly associated to other cuproptosis‐related mediators (0.19 < |R| < 0.80 and *p* < 0.05). Overall, these findings imply that crosstalk between Cu metabolism‐associated genes is present in COPD development. Furthermore, we constructed a WGCNA network using expression profile data from GSE20257 to explore the co‐expression network related to COPD. Among the 15 identified modules in the GSE20257 dataset, the black module (comprising 1018 genes) displayed a high correlation with the COPD phenotype (*R* ^2^ = 0.56, *p* = 6e^−11^). Subsequently, we overlapped the genes from the black module of GSE20257 with Cu metabolism‐associated genes, resulting in the identification of five common genes (see Figure S2).

### 3.3 Correlation between copper metabolism‐related genes and COPD key genes

**FIGURE 3:**
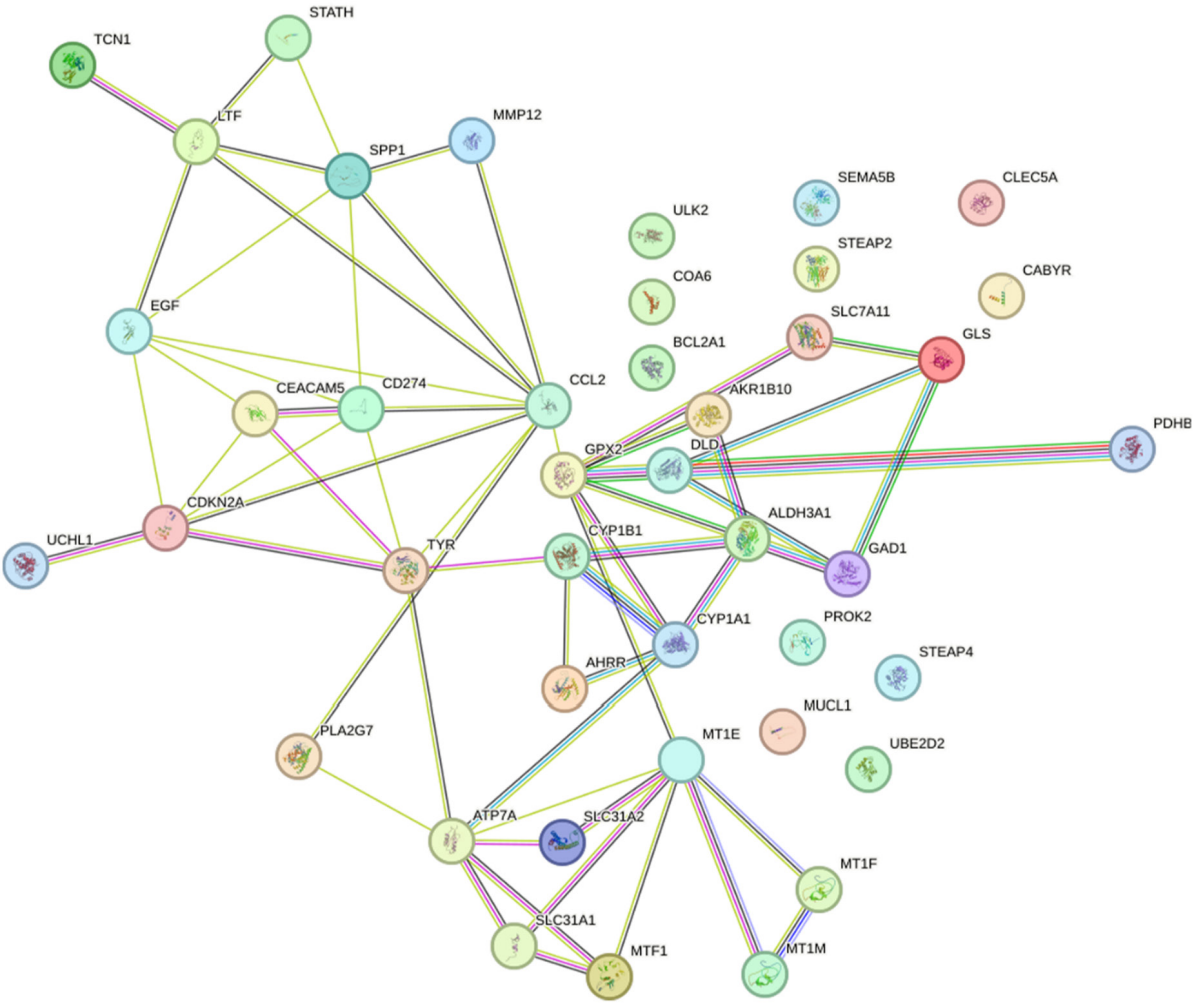
Protein–protein interactions among 18 copper metabolism‐related genes and 27 key COPD genes.

In a previous report, 27 key COPD genes were screened.^25^ We performed PPI interaction analysis between those 27 key genes and 18 copper metabolism‐related genes. In the PPI network analysis, we observed an interaction between the copper metabolism‐related gene DLD and a crucial COPD gene, GPX2, which belongs to the glutathione peroxidase family (GPXs, GSH‐Px). Previous research has reported a negative correlation between muscle EGF transcription levels and ST fibre proportion in COPD patients,^26^ and our PPI results indicated an interaction between CDKN2A and EGF. Notably, CDKN2A exhibited potential interactions with several key COPD genes (see Figure 3). These findings suggest that the copper metabolism‐related genes within these modules are closely associated with COPD.

### 3.4 Bioinformatics analysis

**FIGURE 4:**
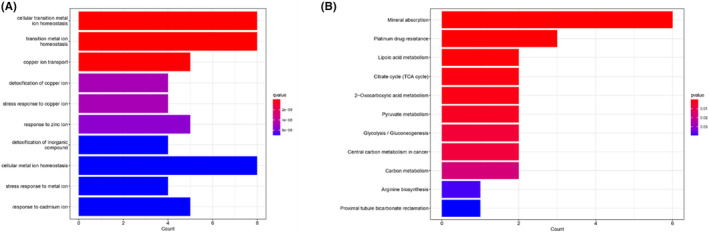
GO and KEGG signalling pathway analyses of the 18 copper metabolism‐related genes. Gene Ontology designations (A) and KEGG pathways generated (B) via DAVID functional annotation. Only top 10 terms were considered to be of significance.

GO and KEGG analyses were conducted for the copper metabolism‐related 18 DEGs to determine possible biological processes related to COPD. GO analysis demonstrated that these genes were enriched in cellular transition metal ion homeostasis, transition metal ion homeostasis, copper ion transport, detoxification of copper ion, stress response to copper ion, response to zinc ion, detoxification of inorganic compound, cellular metal ion homeostasis, stress response to metal ion, response to cadmium ion, implying that these genes are involved in the occurrence of COPD by affecting these molecular functions and biological processes (Figure 4A). KEGG analysis indicated that the mineral absorption, platinum drug resistance, lipoic acid metabolism, citrate cycle (TCA cycle), 2‐oxocarboxylic acid metabolism, pyruvate metabolism, glycolysis/gluconeogenesis, central carbon metabolism in cancer, carbon metabolism which influence the ability of the lung and the susceptibility to lung disorders, are related to COPD (Figure 4B).

### 3.5 Lung function and histopathology in COPD model rats

**FIGURE 5:**
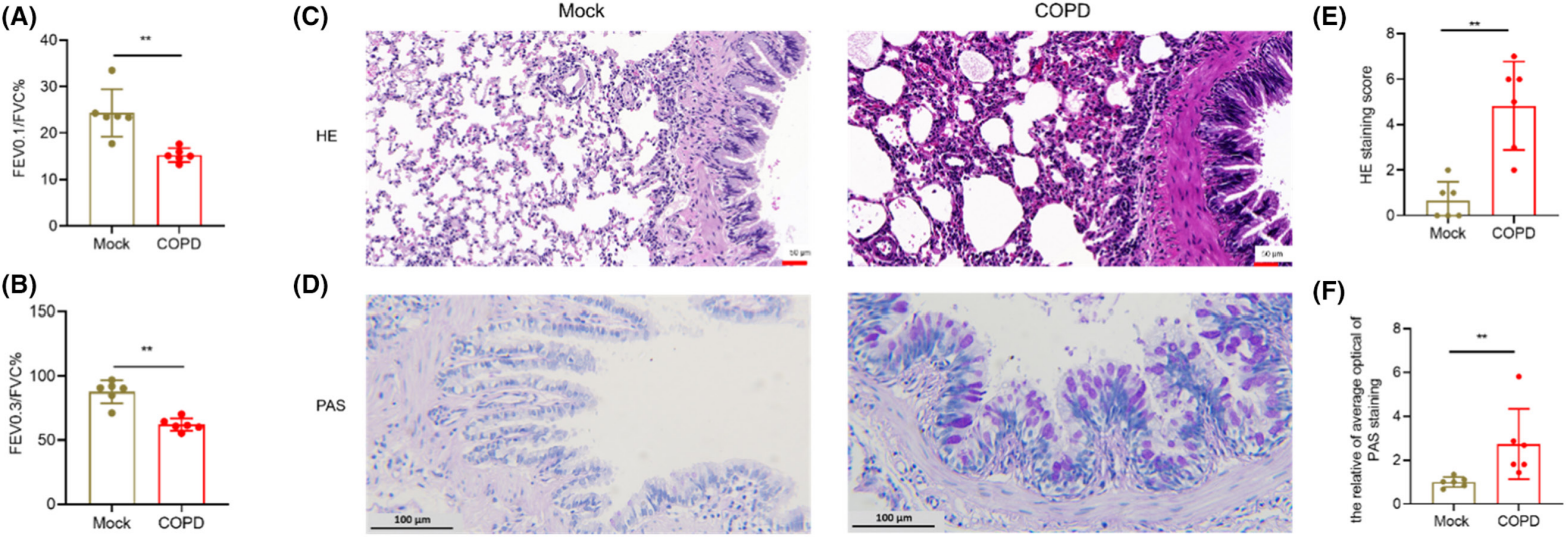
Lung function and histopathology in COPD rats. (A, B) Lung function FEV0.1/FVC % (A) and FEV0.3/FVC % (B) measured after 12 weeks; (C, D) HE staining (C) and PAS staining (D) in lung tissue of COPD rats; (E‐F) HE staining scores (E) and PAS staining average optical (AO) values (F) of mock and COPD groups. *p < 0.05, **p < 0.01.

To further verify the regulatory roles of cuproptosis and Cu metabolism‐associated genes, we used rats to build COPD models. Lung function can reflect airflow conditions of rats with COPD. Compared to control group, the main lung function indices in COPD group were decreased for FEV0.1/FVC (*p* < 0.01) and FEV0.3/FVC (*p* < 0.01) (Figure 5A,B). In the lung of COPD group rats, the alveolus septum appeared thickened, with oedema and inflammatory cell infiltration. Pulmonary inflammatory score and damage index of COPD rats were higher than those of the mock group (Figure 5C,E). The goblet in the tracheal epithelium was stained with PAS. The PAS positive staining areas were examined and analysed as mucus secretion and goblet metaplasia. Histological assessment showed an increase in proliferation of goblet cells and mucus blockage induced by CS in COPD rats (Figure 5D,F).

### 3.6 Increased copper and abnormal expression of copper regulatory factors in lung tissue of COPD model rats

**FIGURE 6:**
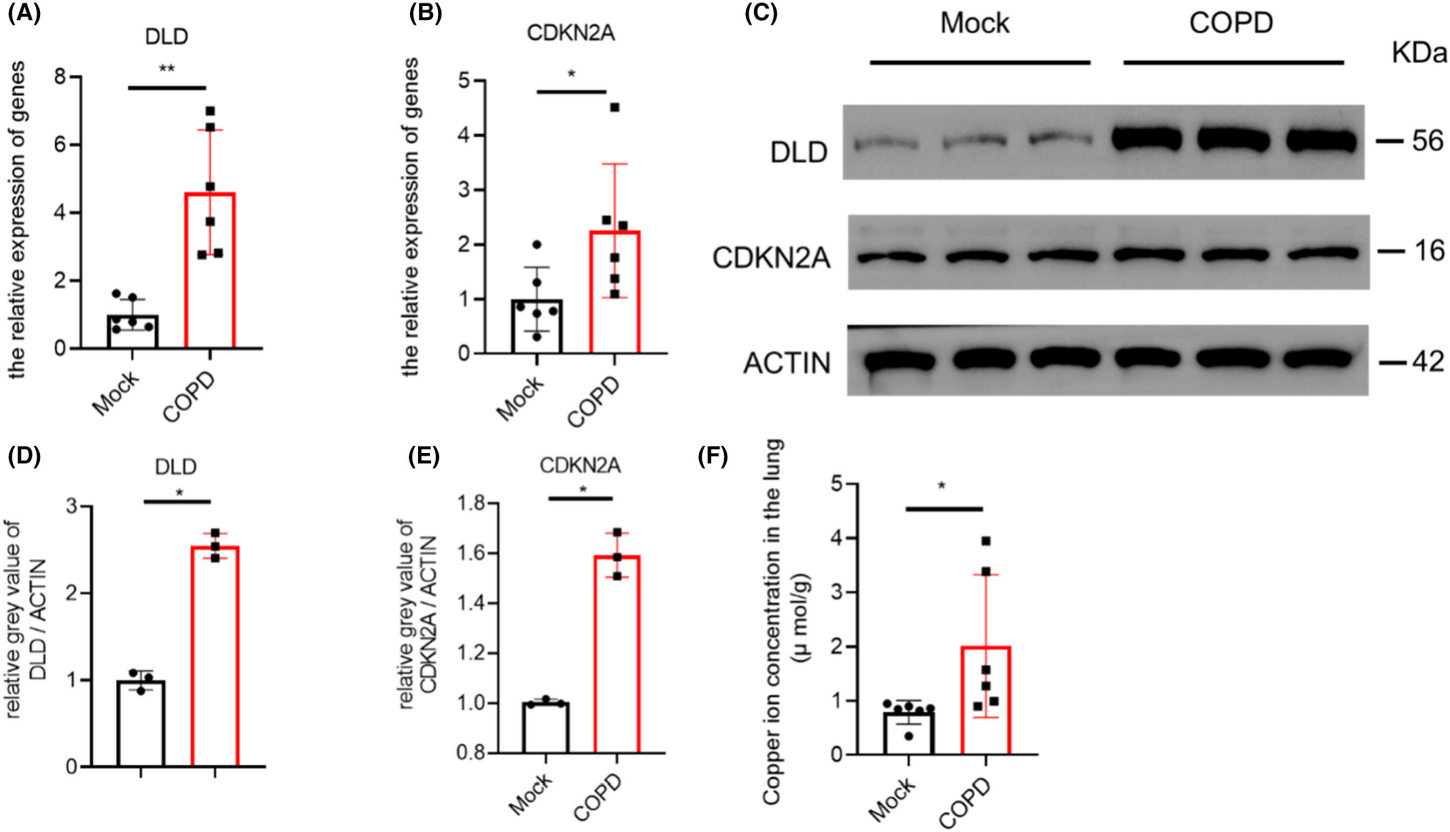
RT‐qPCR and western blot analysis of cuproptosis genes DLD and CDKN2A. (A, B) RT‐qPCR assays of DLD and CDKN2A. Fold‐change in expression is shown relative to control groups (*n* = 6 per group); (C) Western blot for detecting the protein expression of DLD and CDKN2A in rat lung tissues; (D, E) analysis relative fold of indicated protein grey level. (F) Copper ion concentration in the rats' lung sample (μ mol/g). *p < 0.05, **p < 0.01.

Subsequently, based on the above‐mentioned sequencing data of COPD clinical patients, we established a rat model of COPD and detected some key regulators involved in cuproptosis, which included DLD and CDKN2A. mRNA expression of these two genes was increased in the model group (Figure 6A,B), and their protein levels also increased significantly (Figure 6C–E) and original image of blots and gels shown in Figure S1. We also found enhanced accumulation of copper in lung tissues of COPD rats (Figure 6F). In conclusion, abnormal accumulation of copper and abnormal expression of copper regulatory factors predict that cuproptosis may occur in COPD model rats.

### 3.7 Effect of smoking and different stages of COPD on expression of cuproptosis‐related genes DLD and CDKN2A

**FIGURE 7:**
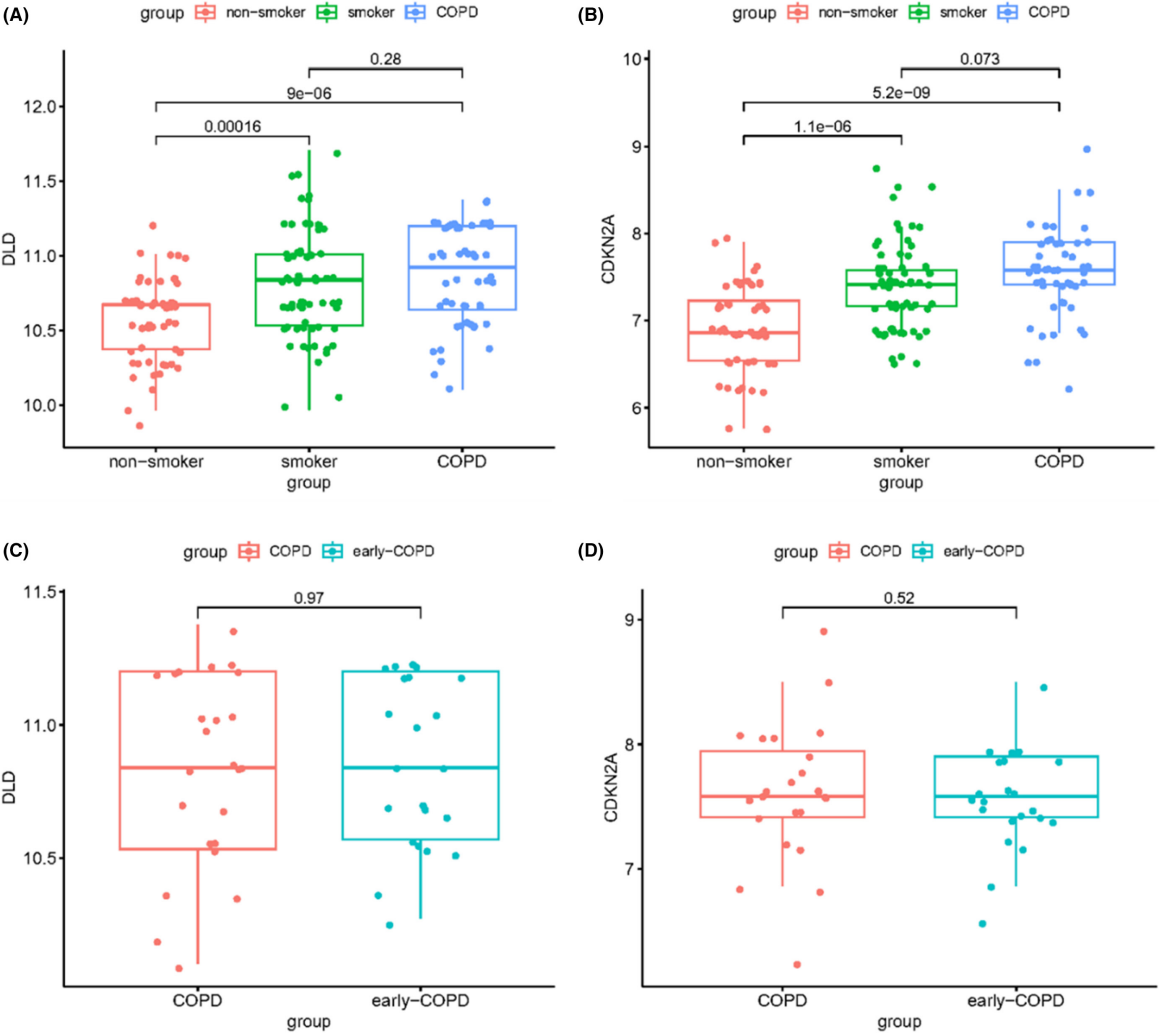
Expression of cuproptosis‐related genes DLD and CDKN2A. (A, B) Expression in healthy non‐smoker, healthy smoker and COPD patients; (C, D) expression in early COPD versus COPD.

To further investigate the correlation between copper cuproptosis‐related genes and COPD, we focused on two important factors in COPD: (1) smoking (healthy or COPD) and non‐smoking (healthy) and (2) early COPD versus established COPD. For analysis, we selected two key cuproptosis‐related genes, DLD and CDKN2A. Our results showed a significant difference in their expression between healthy smokers and healthy non‐smokers (*p* < 0.05). Their expression levels in the COPD patient group were the highest, but there was no significant difference in expression levels when compared to healthy smokers (*p* > 0.05) (Figure 7A,B). When comparing their expression levels between early‐stage COPD and established COPD patients, no significant differences were found (*p* > 0.05) (Figure 7C,D). These suggest that the expression of these cuproptosis‐related genes does not change significantly with COPD progression. In summary, our results suggest that smoking has some promoting effect on increasing the expression of these genes, but no differences in expression levels were found between early‐stage and established COPD groups.

## 4 DISCUSSION

Abnormal metal accumulation disrupts cellular homeostasis and leads to disease. Indeed, cuproptosis regulates metabolic pathways, mitochondrial metabolism and immune microenvironment,^27^, ^28^, ^29^, ^30^, ^31^ but how cuproptosis affects COPD is still unknown. Thus, we have explored the integrated role of copper metabolism‐related genes in COPD. An imbalance in copper homeostasis can lead to oxidative damage. Although a positive association between copper and chronic lung disease has been reported, the underlying cause remains unclear. Sputum copper levels were elevated in patients with lung disorders, especially COPD and asthma.^32^ Increase in copper in COPD patients may be due to excessive inhalation of PM2.5,^33^, ^34^ an increase in accumulation in the body because of a reduced excretion caused by smoking,^35^ or to an excessive intake of copper in special environments, for example, in welding workers.^36^, ^37^ Gender, cardiovascular disease, COPD, lung disease clinical stage and haemoglobin, platelet and glucose levels markedly distinguish copper and zinc status.^38^ All‐cause mortality in lung cancer patients is positively related to serum copper level, copper:zinc ratio and whole blood zinc level. Nevertheless, advanced clinical stages of the disease are the most significant predictor of all‐cause mortality. The circulating status of copper and zinc may be considered as additional prognostic variables in routine clinical features of lung cancer patients.^38^

Protein lipoylation are important factors of cell death induced by copper. As DLD is a key enzyme in the lipoic acid pathway, knocking out this gene can significantly alleviate copper ion carrier‐mediated cytotoxicity.^18^ The typical aging marker p16 (CDKN2A) is one the most expressed genes in aging lungs. Through a comprehensive analysis of multiple omics data from over 10,000 samples across 33 tumour types, it was found that CDKN2A is the copper death regulator with most frequent occurrences of somatic mutations.^39^ Lung cancer and COPD often co‐occur, and individuals with COPD are at a higher risk of developing lung cancer. Therefore, CDKN2A may play a crucial role in inhibiting the progression from COPD into lung cancer. In further, our results showed that smoking has some promoting effect on increasing expression of cuproptosis‐related genes DLD and CDKN2A. Excess of Cu is toxic by inducing oxidative stress, and it induces crosstalk between mitophagy and apoptosis.^40^ In in vivo experiments, feeding chickens with different levels of copper resulted in ultrastructural damage, mitophagy and apoptosis in liver tissue. In primary chicken hepatocytes, copper exposure induced mitophagy via the Parkin/PINK1 pathway, and mitophagy may ameliorate Cu‐induced mitochondrial apoptosis.^40^ An excess of copper can induce the production of lactate dehydrogenase (LDH), increase ROS, superoxide dismutase (SOD), decreased glutathione (GSH) activity and MMP, dose–response upregulated Bax, Bak1, caspase3 and CytC mRNA and induce apoptosis. NAC treatment alleviated the Cu‐induced changes of all of the above factors.^41^ These results suggest that excess copper can trigger oxidative stress and cell death in chicken hepatocytes via the mitochondrial pathway. Therefore, the possibility that cuproptosis is synergistic with other copper‐induced programmed cell death pathways may constitute a novel research direction.

In conclusion, our study demonstrates that DLD and CDKN2A are expressed in COPD, with functional roles as cuproptosis mediators. Their expression is abnormal in the airway epithelial cells of COPD patients, and smoking has some promoting effect on increasing their expression. This has been verified in COPD model rats. COPD occurrence is linked to copper regulation genes. How these genes are regulated and whether the abnormal interactions among them contribute to the development of COPD still need to be further explored.

## AUTHOR CONTRIBUTIONS

**Wenchuan Qi:** Conceptualization (equal); data curation (lead); formal analysis (equal); funding acquisition (equal); methodology (lead); software (equal); validation (equal); visualization (equal); writing – original draft (lead); writing – review and editing (equal). **Lu Liu:** Conceptualization (equal); data curation (equal); formal analysis (equal); methodology (equal); software (equal); writing – original draft (equal); writing – review and editing (equal). **Qian Zeng:** Conceptualization (equal); data curation (equal); formal analysis (equal); methodology (equal); software (equal); writing – original draft (equal); writing – review and editing (equal). **Ziyang Zhou:** Data curation (equal); investigation (equal); methodology (equal); visualization (equal). **Daohong Chen:** Data curation (equal); investigation (equal); methodology (equal); visualization (equal). **Bin He:** Data curation (equal); methodology (equal); visualization (equal). **Siyao Gong:** Methodology (equal); supervision (equal); validation (equal). **Lei Gao:** Data curation (equal); software (equal). **Xiao Wang:** Methodology (equal); visualization (equal). **Jian Xiong:** Methodology (equal); visualization (equal). **Dingjun Cai:** Conceptualization (equal); resources (equal); visualization (equal); writing – review and editing (equal). **Shuguang Yu:** Funding acquisition (lead); project administration (equal); writing – review and editing (equal). **Ling Zhao:** Funding acquisition (lead); project administration (lead); writing – review and editing (lead).

## FUNDING INFORMATION

This research was funded by National Natural Science Foundation of China (No. 82205286), Innovation Team and Talents Cultivation Program of National Administration of TCM (No: CXZYYTD‐D‐202003), Sichuan Provincial Department of Science and Technology Central Leading Local Science and Technology (No. 2021ZYD0103) and the Natural Science Foundation of Sichuan Province (No. 2023NSFSC1819).

## CONFLICT OF INTEREST STATEMENT

The authors declare that they have no competing interests.

## Supporting information


Figure S1.
Figure S2.

Table S1.

Table S2.

## Data Availability

The data supporting the findings of this research are available from the corresponding author upon request.
