# Supplementary material for: Contribution of cuproptosis and Cu metabolism‐associated genes to chronic obstructive pulmonary disease
Source: J Cell Mol Med. 2023 Oct 6;27(24):4034–44. doi: 10.1111/jcmm.17985 (PMC10747414; doi:10.1111/jcmm.17985)
Supplement: Supplementary file 1 — Figure S1. Figure S2. [file JCMM-27-4034-s002.pdf]

Supplementary Figure S1 Original image of blots and gels in the article

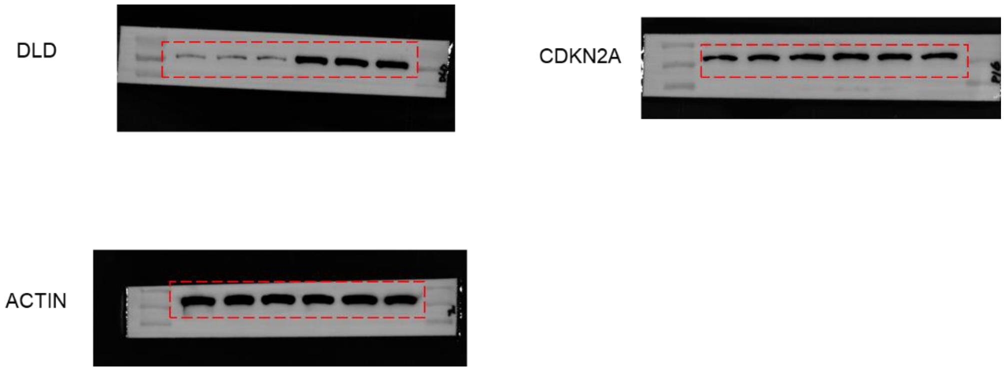

Figure S1. The original blots were used in Figures 6C-D, please note the blots cut prior to hybridization with antibodies.

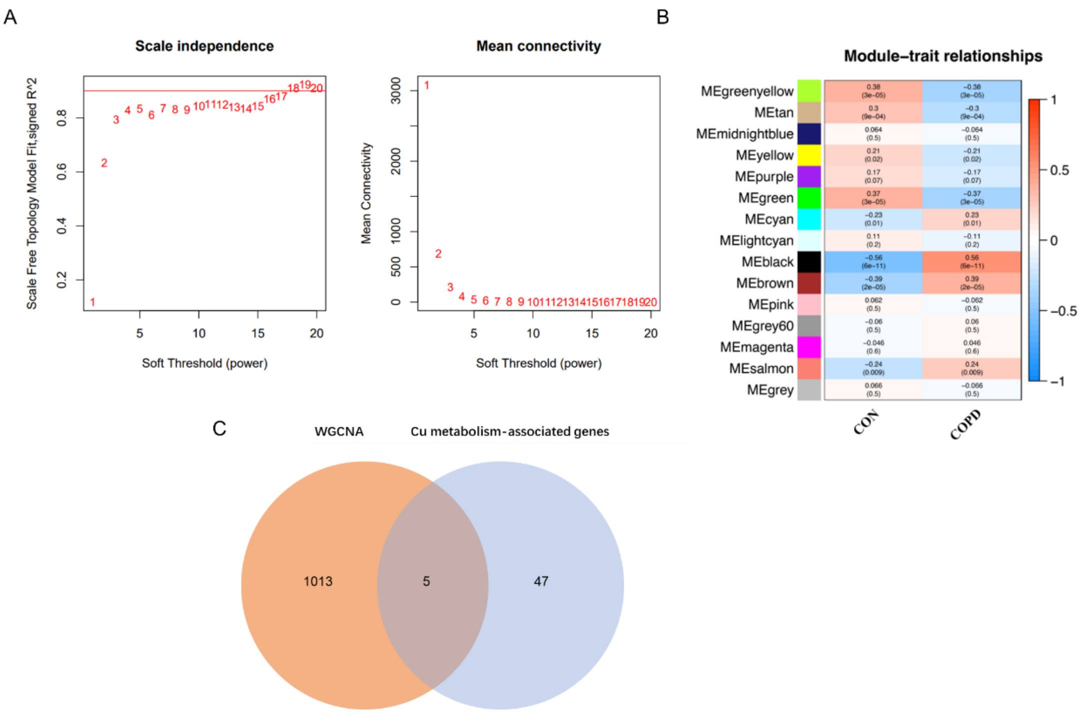

Figure S2. WGCNA for co-expression network construction of GSE20257. (A) Determination of soft-thresholding power for GSE20257. (B) Heatmap of the correlation between module eigengenes and the occurrence of COPD. (C) Black module of GSE20257 was intersected with the Cu metabolism-associated genes. Modules with different colors represent different gene modules, and the numbers in the modules represent the correlation between the module and the phenotype.
